# Corrigendum: Trends and Inequalities in Unplanned Pregnancy in Three Population-Based Birth Cohorts in Pelotas, Brazil

**DOI:** 10.3389/ijph.2021.1604257

**Published:** 2021-08-19

**Authors:** Laísa Rodrigues Moreira, Fernanda Ewerling, Iná S. dos Santos, Fernando César Wehrmeister, Alicia Matijasevich, Aluisio J. D. Barros, Ana M. B. Menezes, Helen Gonçalves, Joseph Murray, Marlos R. Domingues, Mariângela Freitas Silveira

**Affiliations:** ^1^ Postgraduate Program in Epidemiology, Federal University of Pelotas, Pelotas, Brazil; ^2^ International Center for Equity in Health, Federal University of Pelotas, Pelotas, Brazil; ^3^ Department of Preventive Medicine, Faculty of Medicine FMUSP, University of São Paulo, São Paulo, Brazil

**Keywords:** unplanned pregnancy, family planning, reproductive health, socioeconomic factors, health inequalities

In the original article, there were errors. A correction has been made to:

The legend in Figure 1 is inverted. The darker dots, that generally have lower prevalence of unplanned pregnancy, represent the women with higher family income; and the lighter dots represent those with lower family income. Unfortunately, this mistake misleads the interpretation of the figure. This error occurred during the final step of the Editorial process, when the authors replaced the color figure with a black and white version.

**FIGURE 1:**
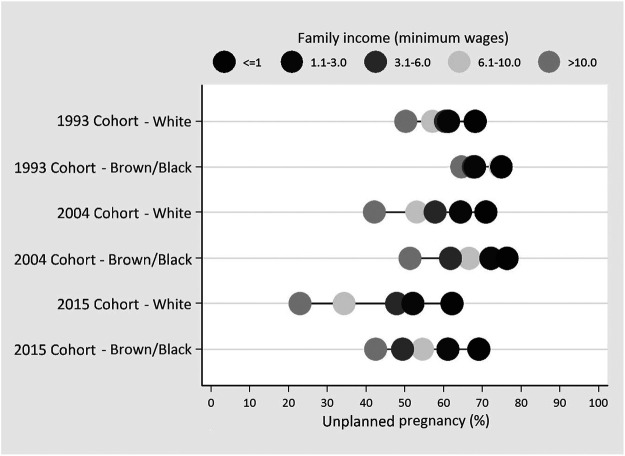
Prevalence of unplanned pregnancy according to monthly family income (in minimum wages) and maternal skin color in three Pelotas Birth Cohorts (Pelotas, Brazil, 1993, 2004, 2015).

Also, in the presentation of the results of Table 3, a minus sign is wrongly placed. Where it reads: “In 1993, the SII was–14.96, i.e., the prevalence of the outcome **was–14.96** percentage points higher among the poorest mothers than among the richest mothers”, it should actually say “In 1993, the SII was–14.96, i.e., the prevalence of the outcome **was 14.96** percentage points higher among the poorest mothers than among the richest mothers”.

I apologize for these mistakes on behalf of all co-authors.

